# Liver Lobe Volumes and the Ratios of Liver Lobe Volumes to Spleen Volume on Magnetic Resonance Imaging for Staging Liver Fibrosis in a Minipig Model

**DOI:** 10.1371/journal.pone.0079681

**Published:** 2013-11-06

**Authors:** Hang Li, Tian-wu Chen, Xiao-ming Zhang, Zhen-lin Li, Jin-ling Zhang, Dan Wang, Ting Li, Jian-lin Wu, Xing Guo, Xiao-li Chen, Li Li, Xian-yong Xie, Zi-shu Zhang

**Affiliations:** 1 Sichuan Key Laboratory of Medical Imaging, and Department of Radiology, Affiliated Hospital of North Sichuan Medical College, Nanchong, Sichuan, China; 2 Department of Radiology, Sichuan Academy of Medical Sciences & Sichuan Provincial People's Hospital, Chengdu, Sichuan, China; 3 Department of Radiology, West China Hospital of Sichuan University, Chengdu, Sichuan, China; 4 Department of Radiology, 2nd Affiliated Hospital of Harbin Medical University, Harbin, Heilongjiang, China; 5 Department of Radiology, Affiliated Zhongshan Hospital of Dalian University, Dalian, Liaoning, China; 6 Department of Ultrasonography, Affiliated Hospital of North Sichuan Medical College, Nanchong, Sichuan, China; 7 Department of Pathology, Affiliated Hospital of North Sichuan Medical College, Nanchong, Sichuan, China; 8 Department of Radiology, University of Michigan Health System, Ann Arbor, Michigan, United States of America; Wayne State University, United States of America

## Abstract

**Objective:**

To investigate liver lobe volumes and the ratios of liver lobe volumes to spleen volume measured with magnetic resonance imaging (MRI) for quantitatively monitoring and staging liver fibrosis.

**Methods:**

Animal study was approved by Institutional Animal Care and Use Committee. Sixteen minipigs were prospectively used to model liver fibrosis, and underwent abdominal gadolinium-enhanced MRI on 0, 5^th^, 9^th^, 16^th^ and 21^st^ weekend after modeling this disease staged by biopsy according to METAVIR classification system. On MRI, volume parameters including left lateral liver lobe volume (LLV), left medial liver lobe volume (LMV), right liver lobe volume (RV), caudate lobe volume (CV), and spleen volume (SV) were measured; and LLV/SV, LMV/SV, RV/SV and CV/SV were calculated. Statistical analyses were performed for staging this fibrosis.

**Results:**

LLV and CV increased with increasing stage of fibrosis (*r* = 0.711, 0.526, respectively; all *P* < 0.05). RV and LMV increased from stage 0 to 2 and decreased from 2 to 4; and RV/SV decreased from 0 to 1, increased from 1 to 2, and decreased from 3 to 4 (all *P* > 0.05). LLV/SV, LMV/SV and CV/SV decreased from stage 0 to 4 (*r* = -0.566, -0.748 and -0.620, respectively; all *P* < 0.05). LLV, CV, LLV/SV, LMV/SV, RV/SV, and CV/SV could distinguish stage 0–1 from 2–4 and 0–2 from 3–4 (all *P* < 0.05). Among these parameters, LLV and LMV/SV could best classify stage ≥2 and ≥3, respectively (area under receiver operating characteristic curve = 0.893 and 0.946, respectively).

**Conclusion:**

LLV and LMV/SV complement each other in staging liver fibrosis, and both parameters should be used to stage this disease.

## Introduction

Liver fibrosis is a consequence of sustained chronic injury from a variety of causes such as viral hepatitis, and often progresses to cirrhosis or liver failure [1,2]. Accurate staging of liver fibrosis is critically important in treatment decision making of specific antifibrotic therapy, and in discriminating advanced from mild liver fibrosis [3–7]. In detail, patients with liver fibrosis stage ≥2 should receive antifibrotic therapy whereas those with the fibrosis stage ≤1 should not [5]. Liver fibrosis stage ≥3 is demonstrated as advanced fibrosis whereas the fibrosis stage ≤2 is mild or early fibrosis [6,7]. Liver biopsy is a gold standard for identifying fibrosis stage ≥2 or ≥3, but it has inherent disadvantages such as some complications, and inter- and intra-observer variability [8,9]. Noninvasive and reliable methods are being investigated for staging liver fibrosis.

Magnetic resonance imaging (MRI) has played an increasingly important role in the assessment of liver fibrosis because it is a safe, effective and repeatable noninvasive modality [10]. With this scanner, total liver volume (TLV) increased from stage 0 to 2 and decreased from stage 2 to 4, and TLV could identify fibrosis stage ≥2 with sensitivity and specificity of approximate 74% [11]. Liu et al [12] indicated that the ratio of TLV to spleen volume (TLV/SV) is of a significant clinical value in the diagnosis of fibrosis stage ≥3. In addition, a previous study reported that SV increased with the progress of liver fibrosis, and could be used for staging liver fibrosis [13]. Because it is well-known that the volumes of each liver lobe and the spleen change with the increasing stages of liver fibrosis, we wanted to know the volume of which liver lobe and the ratio of which lobe volume to SV could best classify the above-mentioned stages of this disease. To our knowledge, there were no reports focusing on the combination of liver lobe volumes and spleen volume measured on MRI to stage liver fibrosis. The purpose of this study was to investigate liver lobe volumes and the ratios of liver lobe volumes to SV measured with magnetic resonance imaging (MRI) for quantitatively monitoring and staging liver fibrosis.

## Materials and Methods

### Ethics statement

This study was carried out in strict accordance with the recommendations in the Guide for the Care and Use of Laboratory Animals of the National Institutes of Health. The protocol was approved by the Committee on the Ethics of Animal Experiments of North Sichuan Medical College.

### Animal model

Sixteen experimental mature minipigs (6 males, 10 females), weighing between 20.0–24.0 kg, were used in our study to model liver fibrosis. The modeling of fibrosis was induced with carbon tetrachloride (CCl_4_) because CCl_4_ is the toxin widely used for experimental study on this disease [14,15]. According to this modeling method, liver fibrosis was induced by intraperitoneal injection of 40% CCl_4_ dissolved in fat emulsion (0.25 mL/kg body weight) twice a week for 16 weeks, and by feeding 40% CCl_4_ (0.75 mL/kg body weight) mixed with maize flour twice daily for the subsequent 5 weeks because of the peritoneal adhesions resulting from the previous intraperitoneal injection. To minimize the chemical peritonitis involving liver, we chose the left hypogastrium as the intraperitoneal injection position and the injections were stopped two days before each MRI examination. We used 5% alcohol-water mixture as the sole drinking water, and maize flour was taken as the staple food in the 21 weeks because the administration of alcohol in conjunction with CCl_4_ could result in accelerated liver fibrogenesis in a large animal model [16].

### MRI technique

The minipigs were examined on a 1.5 T whole body MRI scanner (Signa; GE Medical Systems, Milwaukee, WI) on 0, 5^th^, 9^th^, 16^th^, and 21^st^ weekends after modeling this fibrosis. As for the preparations of the animals, the intraperitoneal injections of CCl_4_ were paused 2 days before each MRI examination. Before the imaging acquisitions, the animals were given general anesthesia by means of an injection of ketamine (15 mL/kg weight) and diazepam (0.8 mg/kg per hour) through one of the ear veins. Subsequently, the anterior surface of the thorax and abdomen of the animal was shaved to obtain good contact between the skin and respiratory triggering. In addition, we used a belt around the abdomen to reduce the effect of respiratory motion during the acquisitions.

When the respiratory signals were established, the animal was positioned supinely in an 8-channel phased array body coil. The routine MRI sequences included SPGR T1-weighted imaging (T1WI), and fast recovery fast spin echo (FRFSE) T2-weighted imaging (T2WI). Subsequently, 10 mL gadodiamide (Magnevist; Bayer Healthcare, Germany) was intravenously injected via a pressure injector (Spectris MR Injection System; Medrad, Warrendale, PA) at a dose of 3 mL/s for a total of 0.2 mmol per kg of body weight followed by a 20 mL saline solution flush for axial contrast-enhanced three-dimensional liver acquisition with volume acceleration (3D-LAVA). The parameters for SPGR T1WI were as follow: TR = 195 ms, TE = 1.5 ms, field of view (FOV) = 36 × 36 cm, slice thickness = 5.0–8.0 mm, slice gap = 0–1 mm, and matrix = 256 × 192 mm. The parameters for FRFSE T2WI were as follows: TR = 12000 ms, TE = 90 ms, FOV = 36 × 36 cm, slice thickness = 5.0–7.0 mm, slice gap = 0.5–1.0 mm, and matrix = 256 × 192 mm. The parameters for axial 3D LAVA were: TR = 3.9 ms, TE = 1.8 ms, FOV = 34 × 34 cm, slice thickness = 5.0 mm, slice gap = 0, and matrix = 256 × 224 mm. The scanning coverage was from the diaphragm to the inferior border of the spleen to cover the entire liver and spleen.

### Image analysis

**Figure 1:**
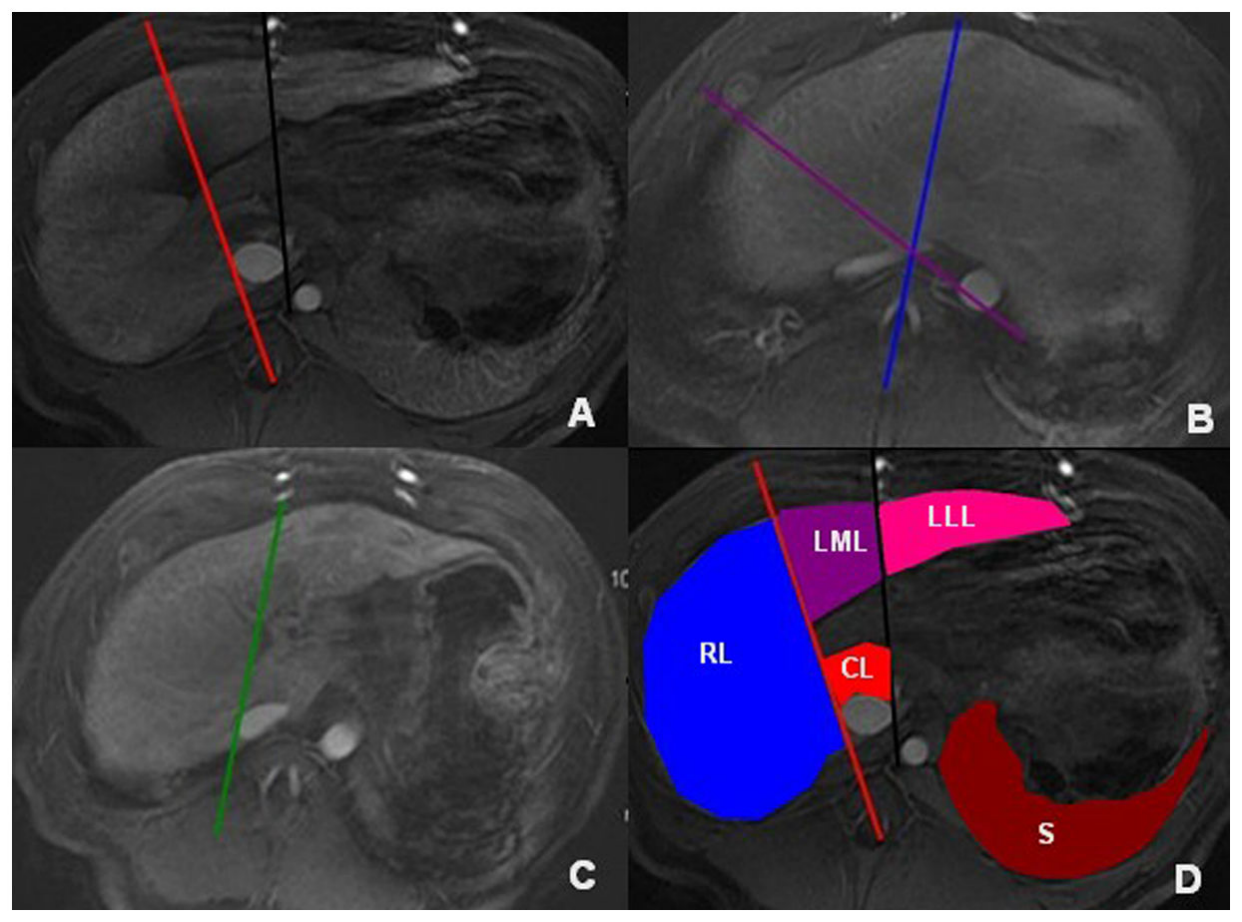
The division of liver lobes. Middle liver fissure (red line), and liver interlobar fissure (black line) are used as a landmark to differentiate right liver lobe from left liver lobe, and left lateral liver lobe from left medial liver lobe on the level of the fossa for gallbladder, respectively (A). Middle liver vein (purple line), and left liver vein (blue line) are used as a landmark to differentiate right liver lobe from left liver lobe, and left lateral liver lobe from left medial liver lobe on the level of the second liver portal, respectively (B). The line (green line) linking the inferior vena cava to the right branch of the portal vein is used as a landmark to differentiate right liver lobe from the caudate lobe on the level of the first liver portal (C). Outlines of four hepatic lobes, and of spleen are delineated on the axial enhanced magnetic resonance imaging (D) (RL, right liver lobe; LML, left medial liver lobe; LLL, left lateral liver lobe; CL, caudate lobe; and S, spleen).

The original MRI data were directly interfaced and forwarded to the workstation (GE, AW4.1, Sun Microsystems, Palo Alto, CA, United States). According to the Goldsmith and Woodburne system, the liver was divided into four lobes: left lateral and medial lobes, right lobe and caudate lobe (Figure 1). Based on the enhanced axial 3D-LAVA images, the portal venous phase images were used for the analysis focusing on measuring each liver lobe volume and SV, which was performed by a radiologist (the first author with 3 years of experience in abdominal radiology) who was blinded to the pathologic results. The liver lobe profile was manually traced excluding the inferior vena cava and gallbladder on each transverse image for measuring each liver lobe volume. The software automatically calculated the number of pixels enclosed by the traced liver lobe contour, and provided the cross-sectional area of the liver lobe on a slice-by-slice basis [17]. This previous process and analysis was repeated for each contiguous transverse level until the entire liver lobe had been covered. Left lateral liver lobe volume (LLV), left medial liver lobe volume (LMV), right liver lobe volume (RV), and caudate lobe volume (CV) were obtained by means of the sum of the corresponding areas multiplied by the section thickness [17]. SV was obtained by a method similar to that of each liver lobe volume measurement. Based on each liver lobe volume and SV, the other liver lobe volume parameters including the ratios of LLV to SV (LLV/SV), LMV to SV (LMV/SV), RV to SV (RV/SV), and CV to SV (CV/SV) were calculated.

Two weeks later, the MRI data of all animals on the 9^th^ weekend were randomly chosen to test the interobserver variability of the measurements. Based on the data, measurements of the above liver lobe volume parameters were performed by an experienced radiologic professor (the corresponding author with 15 years of experience in abdominal radiology). The precision of the two measurements was determined as the coefficient of variation on the basis of the formula: coefficient of variation (%) = (*s*/X) × 100, where *s* is the standard deviation and X is the mean of each liver lobe volume parameter. The resultant precision was expressed as an average coefficient of variation. When the coefficient of variation was less than 10%, interobserver variability of each liver lobe volume parameter measurement was regarded as small, and the first measurement was used as the final liver lobe volume parameter [18]. If the coefficient of variation exceeded 10%, another measurement by the first author was made and an average of the two measurements was used as the final liver lobe volume parameter.

### Histopathology

**Figure 2:**
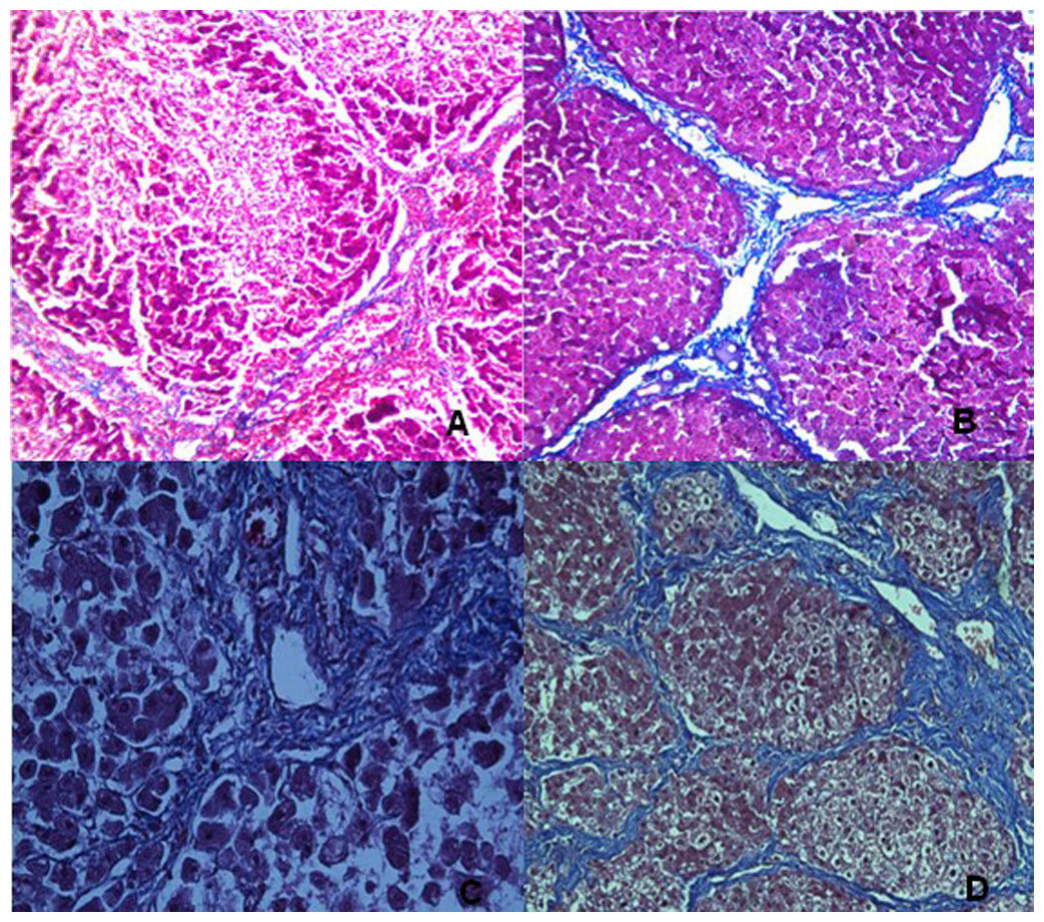
Photomicrographs (original magnification, ×400; Masson’s trichrome stains) of histologic sections from liver biopsy specimens. The figure shows the progression of fibrosis in minipigs: stage 1, portal fibrous expansion (A); stage 2, thin fibrous septa emanating from portal triads (B); stage 3, fibrous septa bridging portal triads and central veins (C); and stage 4, cirrhosis (D).

After the MR examination on each minipig, an 18-gauge ultrasound-guided core percutaneous biopsy was performed in the right liver lobe because the biopsy in this lobe was used as the standard for staging liver fibrosis [19]. When the minipigs died during the follow-up period before 21^st^ weekend, the dead minipigs underwent immediate laparotomy, and the entire liver was resected. When the minipigs lived on 21^st^ weekend, 1/3 surviving animals were randomly sacrificed by air injection into one of the auricular veins shortly after the last percutaneous biopsy and underwent the laparotomy, and the entire liver was also resected. Subsequently, the resected liver was used for further staging liver fibrosis to confirm the fibrosis stage determined by percutaneous biopsy immediately prior to the animals’ death. Hepatic tissue specimens obtained by biopsy and by laparotomy were stained with Masson’s trichrome staining for pathologic examination. Two experienced hepatopathologists (the 11th and 12th authors with 12 and 38 years of experience in hepatopathology, respectively) scored the pathological specimens using the METAVIR classification system [20]. This scoring system has a five-point scale: stage 0, no fibrosis; stage 1, portal fibrosis (Figure 2A); stage 2, periportal fibrosis (Figure 2B); stage 3, septal fibrosis (Figure 2C); and stage 4, cirrhosis (Figure 2D). Additionally, we considered the weekend on which liver fibrosis was initially confirmed by pathologic examination as that the fibrosis occurred during the follow-up.

### Statistical analysis

All statistical analyses were carried out with SPSS (version 17.0, SPSS, Chicago IL, United States). A *P* < 0.05 was considered to represent a significant difference. The interobserver agreement for the two pathologists with regard to the fibrosis stage, or for the above-mentioned percutaneous biopsy and the laparotomy was expressed by means of *k* statistics. When *k* values were 0–0.40, 0.41–0.60, 0.61–0.80, and 0.81–1.00, the concordance was considered as poor, moderate, good, and excellent agreement, respectively.

Spearman’s rank correlation analyses were used to assess the correlations between each liver lobe volume parameter and fibrosis stage because the liver lobe volume parameters in this study were of skewed distribution. The liver lobe volume parameters were compared between participants stratified by fibrosis stages using Mann-Whitney tests together with Bonferroni corrections for multiple comparisons. The cutoff values of the liver lobe volume parameters were then determined with receiver-operating characteristic (ROC) analysis for classifying liver fibrosis stage ≥2 and ≥3.

## Results

### Animal model and histopathologic findings

During the follow-up period, one, two, and four animals died between week 5 and 9, between week 9 and 16, and between week 16 and 21, respectively. On 0, 5^th^, 9^th^, 16^th^, and 21^st^ weekend, mean body weight of the surviving animals is illustrated in Table 1. There was no significant difference in body weight between any two weekends during the follow-up period (all *P* > 0.05).

**Table 1:** Body weight and liver fibrosis stages of surviving miniature minipigs on the follow-up weekends.

Weekend (*n*)	Body weight (kg)	S0	S1	S2	S3	S4
0 (16)	22.0 ± 1.31	16	0	0	0	0
5 (16)	24.1 ± 2.41	3^†^	8	5	0	0
9 (15)	24.0 ± 2.45	0	3	7	5	0
16 (13)	25.3 ± 2.81	0	1^†^	1	7	4
21 (9)	23.5 ± 1.78	0	1^†^	1^†^	1	6

Notes: ^†^ Animals with liver fibrosis at the current stage are same as those at the immediately previous stage during the follow-up. S0, S1, S2, S3 and S4 represent stage 0, 1, 2, 3 and 4, respectively.

According to the METAVIR classification system, surviving minipigs with liver fibrosis at stages confirmed on the follow-up weekends are illustrated in Table 1. There was excellent agreement between the two independent pathologists with regard to staging fibrosis (*k* = 0.85; 95%CI, 0.79–0.89). In addition, there was good agreement between fibrosis stages determined by laparotomy and by the last percutaneous biopsy for the dead animals before 21st weekend and the randomly killed animals on 21st weekend after modeling the fibrosis (*k* = 0.80; 95%CI, 0.75–0.84).

### Interobserver variability of each liver lobe volume parameter measurements

In all surviving animals on 9^th^ weekend after modeling liver fibrosis, the numbers of minipigs with coefficient of variations less than 10% and exceeding 10% are shown in Table 2. The mean coefficient of variation for each liver lobe volume parameter measurements is also illustrated in Table 2.

**Table 2:** Interobserver variability of each liver lobe volume parameter between two measurements on 9th weekend.

Liver lobe volume	Mean coefficient of	Coefficient of	Coefficient of
Parameters	variation (range)	variation ≤ 10% (n)	variation > 10% (n)
LLV (cm^3^)	9.4% (1.6%–17.7%)	11	4
LMV (cm^3^)	9.6% (3.3%–16.1%)	12	3
RV (cm^3^)	8.5% (2.4%–15.9%)	13	2
CV (cm^3^)	8.7% (3.3%–16.1%)	12	3
LLV/SV	7.3% (1.4%–15.8%)	13	2
LMV/SV	7.7% (1.4%–14.5%)	13	2
RV/SV	6.7% (0.8%–11.6%)	14	1
CV/SV	6.5% (1.3%–11.3%)	14	1

Notes: LLV, left lateral liver lobe volume; LMV, left medial liver lobe volume; RV, right liver lobe volume; CV, caudate lobe volume; and SV, spleen volume.

### Liver lobe volume parameters corresponding to stages of liver fibrosis

**Figure 3:**
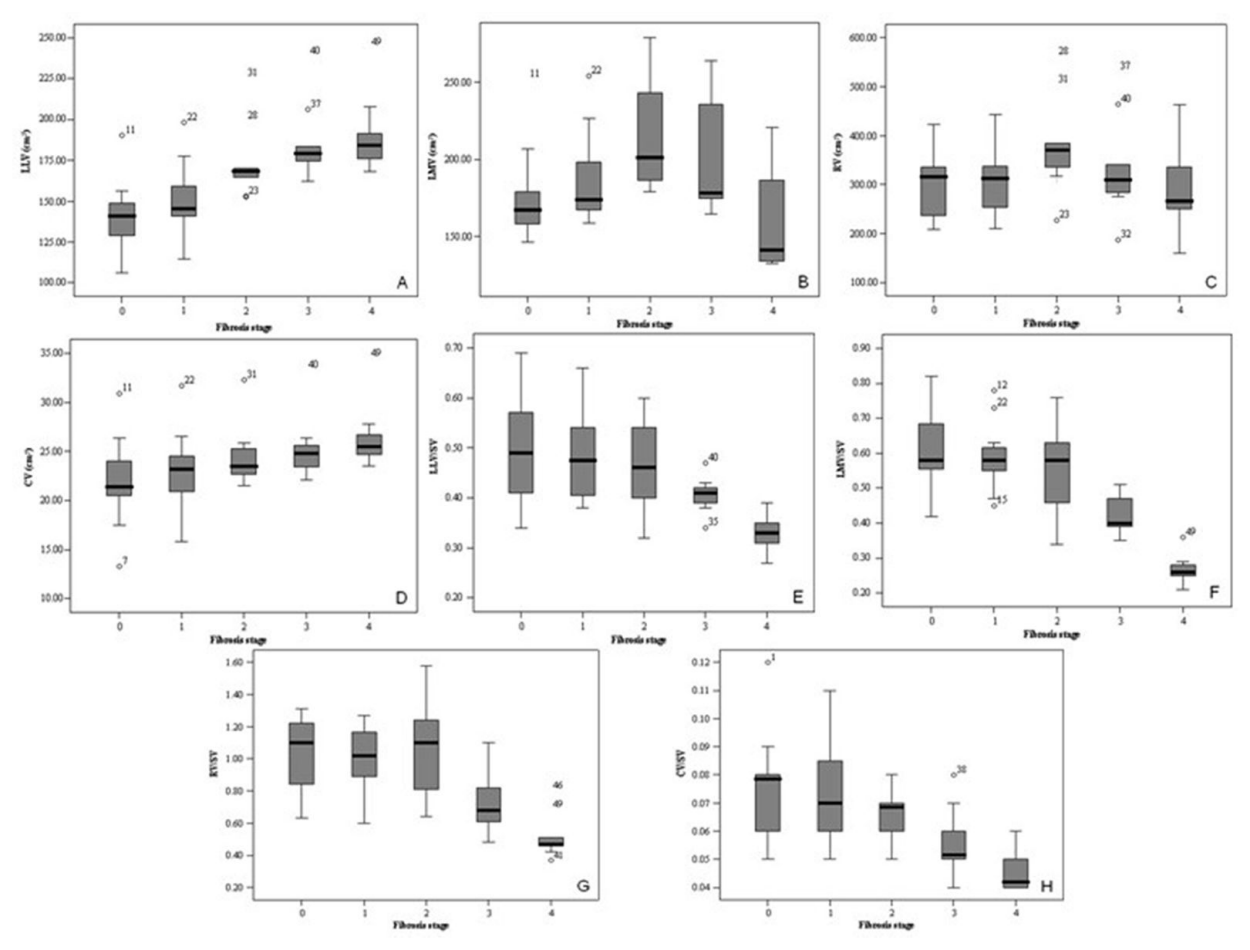
Box plots of liver lobe volume parameters for each fibrosis stage. The figure shows distributions of left lateral liver lobe volume (LLV, A), left medial liver lobe volume (LMV, B), right liver lobe volume (RV, C), and caudate lobe volume (CV, D) as well as the ratios of LLV to SV (LLV/SV, E), LMV to SV (LMV/SV, F), RV to SV (RV/SV, G), and CV to SV (CV/SV, H) stratified by fibrosis stages.

Liver lobe volume parameters corresponding to stages of liver fibrosis are shown in Table 3 and Figure 3. LLV and CV increased from stage 0 to 4 (*r* = 0.711 and 0.526, respectively; all *P* < 0.001). RV and LMV increased from stage 0 to 2, but decreased from stage 2 to 4 (all *P* > 0.05). RV/SV decreased from stage 0 to 1, increased from stage 1 to 2, and decreased from stage 2 to 4 (all *P* > 0.05). LLV/SV, LMV/SV and CV/SV decreased from stage 0 to 4 (*r* = -0.566, -0.748 and -0.620, respectively; all *P* < 0.001). LLV, CV, LLV/SV, LMV/SV, RV/SV and CV/SV could distinguish stage 0–1 from 2–4, and stage 0–2 from 3–4 (all *P* < 0.05). LMV and RV could not distinguish stage 0–1 from 2–4, and stage 0–2 from 3–4 (all *P* > 0.05).

**Table 3:** Liver lobe volume parameters corresponding to stages of liver fibrosis.

Stage (n)	LLV (cm^3^)	LMV (cm^3^)	RV (cm^3^)	CV (cm^3^)	LLV/SV	LMV/SV	RV/SV	CV/SV
0 (16)	141.23 ± 21.62	176.41 ± 30.1	301.41 ± 67.41	22.05 ± 4.6	0.5 ± 0.12	0.62 ± 0.11	1.07 ± 0.24	0.07 ± 0.02
	(106.1–180.4)	(146.6–252.4)	(207.6–402.2)	(13.3–30.9)	(0.34–0.59)	(0.42–0.82)	(0.63–1.31)	(0.05–0.12)
1 (11)	151.53 ± 22.36	186.98 ± 30.72	310.19 ± 68.95	23.1 ± 4.01	0.48 ± 0.09	0.59 ± 0.1	0.99 ± 0.23	0.07 ± 0.02
	(114.6–198.3)	(158.6–254.3)	(209–443.4)	(15.8–31.7)	(0.38–0.66)	(0.47–0.78)	(0.6–1.24)	(0.05–0.11)
2 (13)	174.41 ± 23.57^a^	197.83 ± 37.02	382.93 ± 107.4	24.62 ± 3.23	0.46 ± 0.09	0.56 ± 0.14	1.13 ± 0.31	0.06 ± 0.01
	(152.7–226)	(164.6–264.3)	(247–528.1)	(21.5–32.3)	(0.32–0.4)	(0.34–0.7)	(0.64–1.58)	(0.05–0.08)
3 (13)	185.72 ± 23.42^b^	193.9 ± 36.1	334.61 ± 104.17	25.45 ± 3.37	0.39 ± 0.03^d^	0.42 ± 0.06^b^	0.70 ± 0.19	0.05 ± 0.01
	(162.3–239.1)	(158.8–259)	(187.1–533.2)	(22.1–33.4)	(0.32–0.6)	(0.35–0.51)	(0.48–0.91)	(0.04–0.08)
4 (10)	190.95 ± 23.24^b^	153.66 ± 33.37	302.74 ± 96.02	26.12 ± 3.3	0.35 ± 0.03^c^	0.28 ± 0.05^c^	0.56 ± 0.14^e^	0.04 ± 0.01^f^
	(168.1–244.4)	(121.2–220.6)	(180–483.1)	(23.5–34.5)	(0.31–0.39)	(0.2–0.29)	(0.37–0.81)	(0.04–0.06)
Grouped								
stages (n)								
0–1 (27)	146.38 ± 22.07	181.67 ± 30.17	305.75 ± 66.69	22.55 ± 4.25	0.49 ± 0.11	0.61 ± 0.1	1.01 ± 0.23	0.07 ± 0.02
	(106.1–190.4)	(146.6–254.3)	(207.6–443.4)	(13.3–31.7)	(0.34–0.66)	(0.42–0.82)	(0.6–1.31)	(0.05–0.12)
0–2 (40)	154.52 ± 25.62	186.35 ± 32.52	328.63 ± 86.73	23.14 ± 4.03	0.49 ± 0.1	0.59 ± 0.12	1.03 ± 0.25	0.07 ± 0.01
	(106.1–226)	(146.6–264.3)	(207.6–528.1)	(13.3–32.3)	(0.34–0.4)	(0.34–0.82)	(0.6–1.58)	(0.05–0.12)
2–4 (36)	183.70 ± 23.55^g^	181.79 ± 39.74	341.67 ± 104.1	25.50 ± 3.22^g^	0.40 ± 0.08^g^	0.41 ± 0.14^g^	0.77 ± 0.32^g^	0.06 ± 0.01^g^
	(152.7–244.4)	(164.6–220.6)	(247–483.1)	(21.5–34.5)	(0.31–0.6)	(0.2–0.7)	(0.37–1.58)	(0.04–0.08)
3–4 (23)	188.34 ± 22.78^h^	173.78 ± 39.59	320.23 ± 98.32	25.96 ± 3.23^h^	0.36 ± 0.52^h^	0.34 ± 0.1^h^	0.62 ± 0.19^h^	0.05 ± 0.01^h^
	(162.3–244.4)	(158.8–220.6)	(187.1–483.1)	(22.1–34.5)	(0.31–0.6)	(0.2–0.51)	(0.37–0.91)	(0.04–0.08)

Notes: Data are expressed as mean ± standard deviation, and numbers in the brackets are the ranges of liver lobe volume parameters. ^a^ Different from stage 0, *P* < 0.05; ^b^ different from stage 0, and from stage 1, *P* < 0.05; ^c^ different from stage 0, from stage 1, and from stage 2, *P* < 0.05; ^d^ different from stage 1, *P* < 0.05; ^e^ different from stage 0, and from stage 2, *P* < 0.05; ^f^ different from stage 1, and from stage 2, *P* < 0.05; ^g^ different from stage 0–1, *P* < 0.05; and ^h^ different from stage 0–2, *P* < 0.05. LLV, left lateral liver lobe volume; LMV, left medial liver lobe volume; RV, right liver lobe volume; CV, caudate lobe volume; and SV, spleen volume.

### ROC analyses of liver lobe volume parameters for classifying stage ≥2 and ≥3

**Figure 4:**
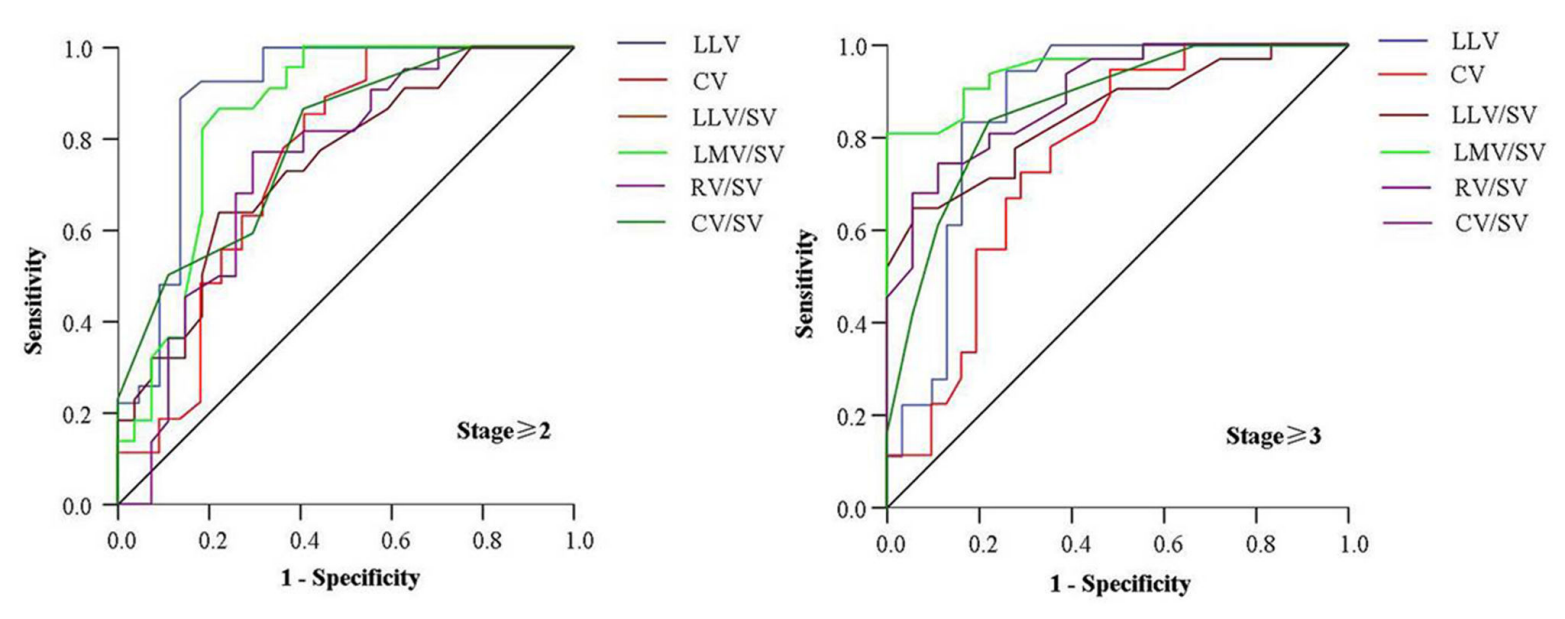
Receiver operating characteristic curves of liver lobe volume parameters for classifying liver fibrosis stage ≥2 and ≥3. The figure shows left lateral liver lobe volume (LLV), caudate lobe volume (CV) as well as the ratios of LLV to spleen volume (LLV/SV), left medial liver lobe volume to spleen volume (LMV/SV), right liver lobe volume to spleen volume (RV/SV), and caudate lobe volume to spleen volume (CV/SV) for classifying liver fibrosis stage ≥2 and ≥3.

In this study, ROC analyses were performed to discriminate stage ≥2 from stage ≤1, and stage ≥3 from stage ≤2 (Figure 4). Table 4 gives the area under the ROC curve (AUC) and the cutoff of liver lobe volume parameters that resulted in both satisfactory sensitivity and specificity for differentiating the previous fibrosis stages. Among all the liver lobe volume parameters, LLV could best classify stage ≥2; and LMV/SV could best classify stage ≥3.

**Table 4:** Receiver-operating characteristic analysis of liver lobe volume parameters for classifying liver fibrosis stage ≥2 and ≥3.

Cutoff	Stage differentiations	AUC	Sensitivity (%)	Specificity (%)
LLV (cm^3^)				
163.53	Stage ≥2	0.893	88.9	86.4
171.54	Stage ≥3	0.865	83.3	83.9
CV (cm^3^)				
23.75	Stage ≥2	0.710	65.4	65.2
24.35	Stage ≥3	0.754	72	71
LLV/SV				
0.410	Stage ≥2	0.742	73.1	63
0.390	Stage ≥3	0.847	77.2	73.3
LMV/SV				
0.525	Stage ≥2	0.848	81.8	81.5
0.415	Stage ≥3	0.946	90	83.3
RV/SV				
0.850	Stage ≥2	0.742	77	68.3
0.725	Stage ≥3	0.866	78.1	76
CV/SV				
0.650	Stage ≥2	0.790	59	71.1
0.550	Stage ≥3	0.860	83.6	78

Notes: LLV, left lateral liver lobe volume; LMV, left medial liver lobe volume; RV, right liver lobe volume; CV, caudate lobe volume; SV, spleen volume; and AUC, area under the receiver operating curve.

## Discussion

 In this study, we found that each liver lobe volume increased from stage 0 to 2, and the possible pathologic mechanism at the early stage would be the ballooning of hepatocytes along with increased fibrotic component [21]. The study showed that LMV and RV decreased from stage 2 to 4, and this finding could be explained by the enlarged range of liver fibrosis and growth of fibrous septa from the expanded portal triad into the surrounding hepatic parenchyma of left medial liver lobe and right liver lobe resulting in atrophy of these liver lobes [22]. Our results were inconsistent with the published report [23] that LMV and RV decreased with the progress of liver fibrosis from stage 0 to 4 when the fibrosis was staged with the Ishak pathologic scoring system. It is the different pathologic scoring system that could explain the inconsistency of our findings with this published article. In addition, this study suggested that LLV and CV continued to increase from stage 2 to 4. We could presume that LLV and CV increased for the compensation of LMV and RV decreasing.

As shown in this study, LLV/SV, CV/SV and LMV/SV decreased from liver fibrosis stage 0 to 4. According to Hoefs et al [24], participants with mild to moderate precirrhotic fibrosis had significant portal hypertension which caused splenomegaly. Due to this portal hypertension, the spleen has been found to undergo architectural and dynamic circulatory alterations including pulp hyperplasia and congestion from increased blood flow in liver fibrosis, which would cause splenomegaly. They also found that a progressive increase in the percentage of SV above the upper limits of normal was noted from no fibrosis to stage 1 (10%), stage 3 (36.7%), and stage 4 (52%). Based on the more and more obvious enlargement of the spleen, we could presume that SV increased more obviously than LLV and CV from stage 0 to 4, leading to the decrease of LLV/SV and CV/SV from stage 0 to 4; and SV increased more obviously than LMV from stage 0 to 2, resulting in the decrease of LMV/SV. The increase of SV and decrease of LMV can lead to the decrease of LMV/SV from stage 2 to 4. This study demonstrated that RV/SV decreased from stage 0 to 1, increased from stage 1 to 2, and decreased from stage 2 to 4. We could presume that SV increased more obviously than RV from stage 0 to 1. With further progress of liver fibrosis from stage 1 to 2, the enlargement of the right liver lobe is more obvious than the enlargement of spleen because of the increase of the ballooning of hepatocytes, which resulted in the increase of RV/SV from stage 1 to 2. Additionally, RV/SV decreased from stage 2 to 4 because RV decreased from stage 2 to 4 and the spleen enlarged with the increasing splenic blood flow [25,26].

In this study, the ROC analysis was performed to classify stage ≥2 and ≥3 based on LLV, CV, LLV/SV, LMV/SV, RV/SV, and CV/SV because of the significant difference in these volume parameters between stage 0–1 and 2–4, and between stage 0–2 and 3–4. As illustrated in this study, the sensitivity and specificity of more than 85% were obtained for classifying stage ≥2 by using the cutoff LLV of 163.5 cm^3^, and the sensitivity and specificity of more than 83% were achieved for classifying stage ≥3 by using the cutoff LMV/SV of 0.415. Compared with other parameters, LLV and LMV/SV could be better indicators for classifying stage ≥2 and ≥3, respectively, because the higher area under the ROC curve, and sensitivity and specificity were obtained. If the two parameters are used to evaluate the treatment effects of liver fibrosis, LMV/SV could potentially be more sensitive to monitor the treatment effects of liver fibrosis stage ≥3 than any other liver lobe parameters whereas LLV could be more sensitive to monitor the treatment effects of stage ≥2.

There were several limitations in this study. Firstly, our sample size was not large. Further studies involving a larger number of samples are needed to evaluate the liver volume parameters for staging liver fibrosis. Secondly, the model used in this study was not a chronic model, but an acute one, and we will perform our further study on whether this method could also be suitable for the chronic cases. Thirdly, our study was not based on a clinical study but on an animal experiment, but our study could provide some useful information that liver lobe volume parameters could be used to stage liver fibrosis. We will conduct our further study to confirm the results in clinical settings.

In conclusion, liver lobe volume parameters obtained by enhanced MRI might be used to monitor the progression of liver fibrosis. LLV and LMV/SV complement each other in staging liver fibrosis, and both parameters should be used to stage this disease. The findings could be helpful for the selection of appropriate liver lobe volume parameter to monitor the treatment effect of liver fibrosis.
